# Expressions of glia maturation factor-β by tumor cells and endothelia correlate with neovascularization and poor prognosis in human glioma

**DOI:** 10.18632/oncotarget.5509

**Published:** 2015-10-26

**Authors:** Xiao-yan Kuang, Xue-feng Jiang, Cong Chen, Xiao-rui Su, Yu Shi, Jin-rong Wu, Peng Zhang, Xin-li Zhang, You-hong Cui, Yi-fang Ping, Xiu-wu Bian

**Affiliations:** ^1^ Institute of Pathology and Southwest Cancer Center, Southwest Hospital, Third Military Medical University, Chongqing, China; ^2^ Key Laboratory of Tumor Immunology and Pathology of Ministry of Education, Southwest Hospital, Third Military Medical University, Chongqing, China; ^3^ Division of Growth and Development and Section of Orthodontics, School of Dentistry, University of California, Los Angeles, CA, USA; ^4^ Collaborative Innovation Center for Cancer Medicine, Sun Yat-sen University, Guangzhou, China

**Keywords:** glia maturation factor-β, glioma, neovascularization, prognosis

## Abstract

Glia maturation factor-β (GMF-β) has been reported to promote glial differentiation, and act as a negative prognostic indicator in certain cancers. However, its roles in glioma progression remain unclear. Since neurogenesis and vasculogenesis were proved to share some common regulators during gliomagenesis, we aim to explore the potential impact of GMF-β on tumor neovascularization and patient survival in glioma. In this study, we first detected GMF-β expression not only in tumor cells but also in microvascular endothelia by double immunohistochemical staining. Both tumoral and endothelial GMF-β expression levels were positively correlated with tumor grade and microvessel density (MVD), while negatively associated with poor prognoses of the patients. Interestingly, multivariate analysis demonstrated that endothelial GMF-β expression level was the only independent predictor of progression-free and overall survival of glioma patients. The results of *in vitro* angiogenesis assay showed that GMF-β knockdown significantly inhibited tubulogenesis of human U87 glioblastoma cells. Furthermore, GMF-β knockdown suppressed tumor growth and the formation of human-CD31 positive (glioma cell-derived) microvessels in a mouse orthotopic U87 glioma model. Our results demonstrated that GMF-β is an important player in glioma progression via promoting neovascularization. GMF-β may therefore be a novel prognostic marker as well as a potential therapeutic target for glioma.

## INTRODUCTION

Glioma is the most frequent type of brain tumors in adults. Stubborn microvascular proliferation is related to high invasiveness and poor prognosis of gliomas [1]. Glioblastoma (GBM, WHO Grade IV) is the most lethal type of malignant glioma, with only a 14-month median survival of patients [2]. Despite recent improvement in antiangiogenic therapy for glioma patients, some vascular-targeting agents brought about therapeutic resistance, owing to complicated mechanisms of tumor neovessel formation [3, 4]. It is in urgent need to identify specific regulators of glioma neovascularization.

Tumor neovessels exhibit heterogeneity both in morphology and in tissue origin [5, 6]. It has been proposed that tumor vessels were mainly derived from both angiogenesis, neovessels sprouting from existing host vessels, and vasculogenesis, neovessels derived from endothelial progenitor cells (EPCs) homing to tumor site [7]. However, it was reported recently that cancer stem cells (CSCs) or glioblastoma cells could generate vascular cells to form new blood channels [8–12]. These findings provide us a new insight into the complicated mechanisms underlying tumor neovascularization.

Tumor neovascularization is often associated with the activation of molecular pathways that regulate embryonic vasculogenesis [7]. It is noted that neural stem cells (NSCs) and endothelial cells (ECs) co-locate in a neurovascular unit during embryogenesis [13]. Moreover, NSCs and ECs are involved in the coordination for reciprocal neurogenesis and vasculogenesis, sharing some common regulators [14, 15]. It has been proved that glioma stem cells (GSCs) and tumor endothelial cells (TECs) also share the same regulating molecules in gliomagenesis, such as vascular endothelial growth factor (VEGF), which enhances neuronal differentiation, and brain-derived neurotrophic factor (BDNF), which promotes ECs survival [16–18].

Glia maturation factor-β (GMF-β) was initially identified as a differentiation inducer for glial cells during nervous system development [19]. GMF-β is predominantly expressed in astrocytes and some neurons. It can promote glial differentiation, neuronal growth, and neural regeneration [20]. In addition, GMF-β was reported to exert a negative feedback control on the proliferation of neural lineage cells [21]. The involvement of GMF-β in tumorigenesis was sporadically reported. Some studies found GMF-β's prodifferentiation effect on glioma, neuroblastoma and medulloblastoma cells *in vitro* [20, 22–23]. Another study showed that GMF-β induced chemosensitivity of glioma cells to cisplatin [24]. In breast cancer and ovarian cancer, GMF-β overexpression was reported to be correlated with poor prognosis [25, 26]. However, the possible roles of GMF-β in tumor neovascularization remain unknown. Therefore, we exerted the effort to elucidate the mechanism of GMF-β underlying glioma neovasculogenesis.

In this study, we examined the expression pattern of GMF-β in human glioma tissues, and assessed its adverse prognostic significance by clinical correlation. Furthermore, we founded that GMF-β plays an important role in inducing the tubulogenesis of glioma cells *in vitro*, as well as the formation of human CD31-positive vessels in a mouse glioma model. So we concluded that the malignant effect of GMF-β in glioma may due to its pro-vasculogenic effect.

## RESULTS

### GMF-β expressions in glioma cells and endothelial cells are positively correlated with both tumor grade and MVD

**Figure 1:**
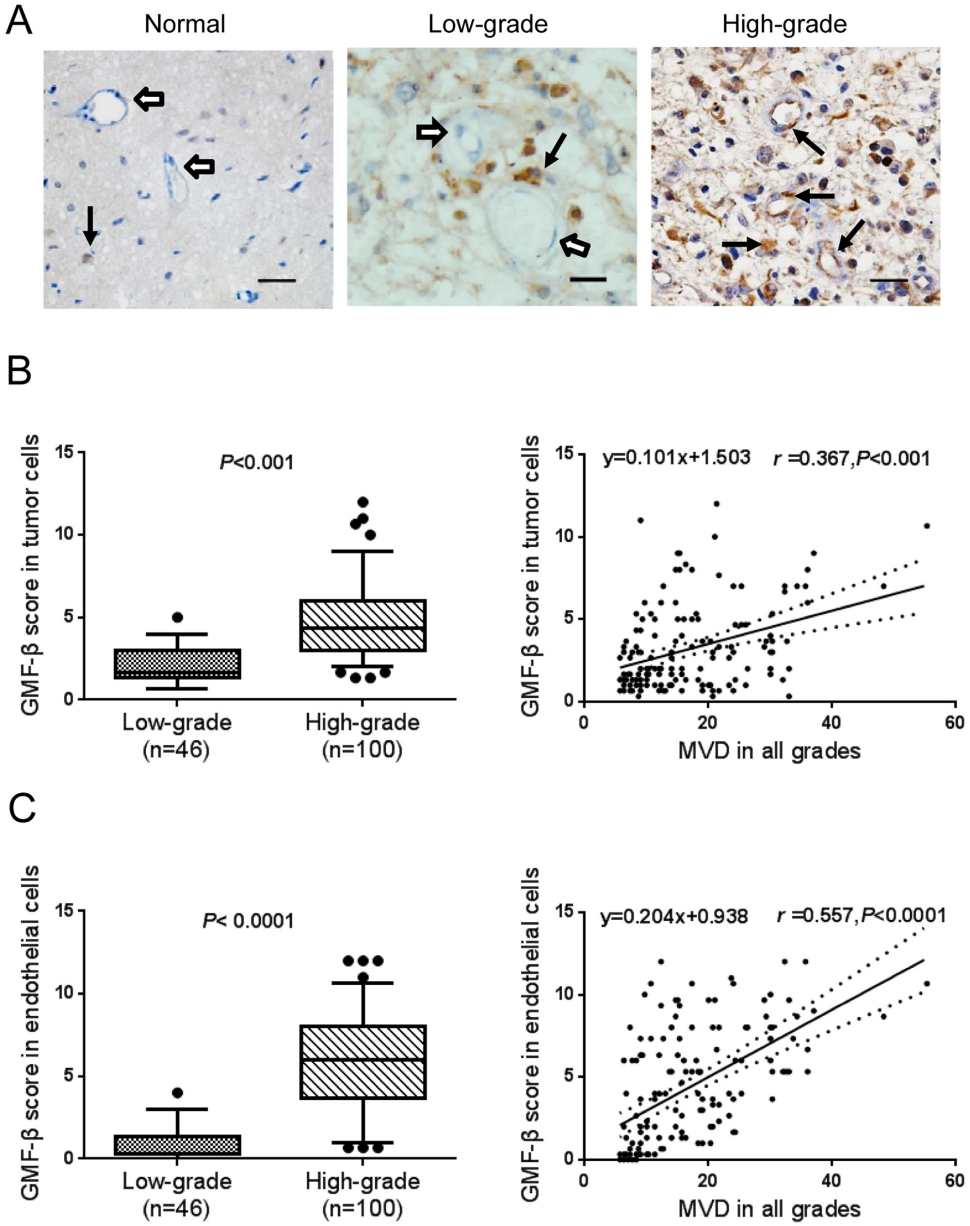
GMF-β expression is positively associated with tumor grade and microvessel density (MVD) in human glioma **A**. Distributions of GMF-β in normal brain tissue (left panel), low-grade (middle panel) and high-grade glioma (right panel). Solid arrows, GMF-β staining (brown); open arrows, negative staining; Scale bar: 50 μm. **B**. IHC-score for GMF-β expression in tumor cells of different grades (left panel); correlation of microvessel densities and tumoral GMF-β expression (right panel). **C**. IHC-score for GMF-β expression in endothelial cells in gliomas of different grades (left panel); correlation of microvessel densities and endothelial GMF-β expressions (right panel).

The expression pattern of GMF-β was examined and analysed by IHC. In normal brain tissues, there were only a few scattered distribution of GMF-β in some glial cells, but absent in blood vessels (Figure 1A, left panel). Over-expressed GMF-β was more frequently found in tumor cells in high-grade gliomas (Figure 1A, right panel) than in low-grade ones (Figure 1A, middle panel). Furthermore, in high-grade gliomas, fairly robust GMF-β immunostaining was dominantly localized in the microvascular endothelial cells (Figure 1A, right panel). In contrast, only few GMF-β expressions were detected in the vessels in low-grade gliomas (Figure 1A, middle panel).

Comparing to low-grade tumor cells, a dramatically higher GMF-β expression was observed in high-grade ones (*P* < 0.001, Figure 1B, left panel). A similar differential expression pattern of GMF-β was obtained in vascular endothelial cells between low-grade and high-grade gliomas (*P* < 0.0001, Figure 1C, left panel). The levels of GMF-β expression were found to be positively correlated with MVD in tumor cells (*r* = 0.367, *P* < 0.001; Figure 1B, right panel), as well as in endothelial cells (*r* = 0.557, *P* < 0.0001; Figure 1C, right panel) in all grades of glioma. These data indicated the association of GMF-β with tumor neovessel formation and stronger pro-vasculogenic potential of GMF-β in higher grade glioma.

### Higher expression of GMF-β is associated with poorer prognosis of glioma patients

**Figure 2:**
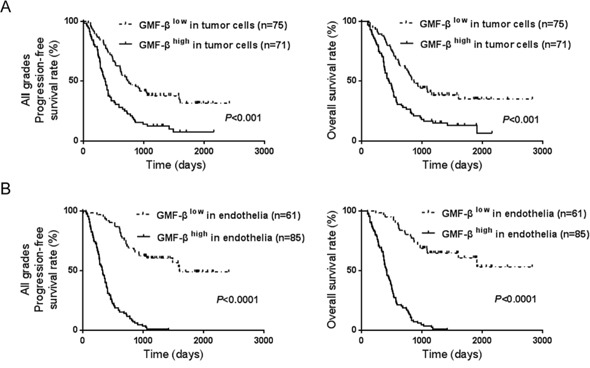
GMF-β expression is negatively correlated with prognoses of glioma patients **A**. GMF-β expression level in tumor cells affects patients’ progression-free survival (left panel) and overall survival (right panel). **B**. Shorter progression-free survival (left panel) and overall survival (right panel) are correlated with high GMF-β in microvascular endothelial cells.

To elucidate the role of over-expressed GMF-β in glioma, we investigated the relationship between GMF-β expression and the clinicopathological features. We first analyzed the correlations of patient survivals with GMF-β expressions status in different tissues of glioma. Kaplan-Meier analysis revealed significant association of higher GMF-β expression in tumor cells with shorter progression-free survival (PFS, Figure 2A, left panel) and overall survival (OS, Figure 2A, right panel) in patients of all WHO grades (*P* < 0.001). Comparing to high GMF-β expression in tumor cells, over-expression of GMF-β in vascular endothelia resulted in much shorter PFS and OS (*P* < 0.0001; Figure 2B).

Subsequently, univariate and multivariate Cox regression analyses were conducted to determine the independence of the prognostic value of GMF-β expression. Univariate survival analysis showed that GMF-β expression in both tumor cells and endothelia were unfavorable prognostic factors for glioma patients (both *P* < 0.0001; Supplementary Table S1). However, multivariate survival analysis verified that GMF-β expression in endothelia was the only independent predictor of both PFS (*P* < 0.0001, *HR* = 1.244, 95% CI = 1.136–1.363) and OS (*P* < 0.0001, *HR* = 1.236, 95% *CI* = 1.126–1.358) in glioma patients (Table 1). These results indicate that over-expressions of GMF-β in both tumor cells and endothelia contribute to poor outcome of glioma patients, in which major effort should be delivered by GMF-β in vascular endothelia.

**Table 1:** Multivariate analyses of progression-free survival and overall survival in glioma patients

*Factors*	*Progression-free survival*		*Overall survival*	
	*HR (95% CI)*	*P-value*	*HR (95% CI)*	*P-value*
WHO grade	3.584 (2.105–6.102)	0.000	3.248 (1.852–5.696)	0.000
Gender	0.779 (0.506–1.200)	0.257	0.794 (0.513–1.230)	0.302
Age	1.012 (0.993–1.031)	0.222	1.013 (0.994–1.032)	0.183
KPS	1.001 (0.984–1.019)	0.889	1.012 (0.993–1.031)	0.216
Ki-67	1.727 (1.253–2.380)	0.001	1.901 (1.356–2.665)	0.000
Resection extent	0.753 (0.491–1.153)	0.192	0.834 (0.534–1.305)	0.427
Chemotherapy	1.668 (1.018–2.732)	0.042	1.632 (0.993–2.683)	0.053
Radiotherapy	1.636 (0.924–2.896)	0.091	1.417 (0.811–2.474)	0.221
Predominant side	0.905 (0.642–1.275)	0.569	0.971 (0.669–1.410)	0.878
Predominant lobe	0.884 (0.758–1.032)	0.120	0.956 (0.813–1.125)	0.586
MVD	0.986 (0.960–1.014)	0.328	0.985 (0.959–1.012)	0.275
*GMF*-β (in endothelia)	1.244 (1.136–1.363)	0.000	1.236 (1.126–1.358)	0.000
*GMF*-β (in tumor cells)	0.980 (0.901–1.065)	0.633	1.020 (0.937–1.111)	0.644

Abbreviations: HR, Hazard ratio; CI, Confidence interval; KPS, Karnofsky performance status; MVD, Microvessel density.

We then analyzed the correlation of GMF-β expression with other clinicopathological parameters. Pearson χ^2^ test indicated that higher GMF-β expression in tumor cells was significantly associated with higher tumor grade (*P* < 0.05) and elevated Ki67 index (*P* < 0.05) (Table 2). On the other hand, higher GMF-β expression in endothelia was closely related to the older age of patients (*P* < 0.001) and higher tumor grade (*P* < 0.001) (Table 3).

**Table 2:** Correlations between clinicopathological parameters and GMF-β expression in tumor cells of glioma

*Parameters*	*GMF-β ^high^ (>2, n = 71)*		*GMF-β ^low^ (≤2, n = 75)*		*P-value*
	*N*	*Percentage (%)*	*N*	*Percentage (%)*	
*Age (years)*					
≥45	35	49	31	41	0.334
<45	36	51	44	59	
*Gender*					
Male	46	65	43	57	0.356
Female	25	35	32	43	
*WHO grade*					
LG	16	23	31	41	0.015
HG	55	77	44	59	
*KPS*					
≥80	40	56	45	60	0.654
<80	31	44	30	40	
*Ki-67 index*					
≥5	47	66	37	49	0.039
<5	24	34	38	51	
*Predominant side*					
L	34	48	31	41	0.585
R	34	48	42	56	
Other	3	4	2	3	
*Predominant lobe*					
Frontal	34	48	35	47	0.192
Temporal	26	37	20	27	
Other	11	15	20	26	

Abbreviations: KPS, Karnofsky performance status; LG, low grade; HG, high grade.

**Table 3:** Correlations between clinicopathological parameters and GMF-β expression in microvascular endothelia of glioma

*Parameters*	*GMF-β ^high^ (>4, n = 67)*		*GMF-β ^low^ (≤4, n = 79)*		*P-value*
	*N*	*Percentage (%)*	*N*	*Percentage (%)*	
*Age (years)*					
≥45	42	63	24	30	<0.001
<45	25	37	55	70	
*Gender*					
Male	44	66	45	57	0.282
Female	23	34	34	43	
*WHO grade*					
LG	0	0	46	58	<0.001
HG	67	100	33	42	
*KPS*					
≥80	36	54	49	62	0.311
<80	31	46	30	38	
*Ki-67 index*					
≥5	27	40	30	38	0.774
<5	40	60	49	62	
*Predominant side*					
L	31	46	34	43	0.714
R	33	49	43	54	
Other	3	5	2	3	
*Predominant lobe*					
Frontal	28	42	41	52	0.014
Temporal	29	43	17	22	
Other	10	15	21	26	

Abbreviations: KPS, Karnofsky performance status; LG, low grade; HG, high grade.

### GMF-β is involved in neovasculogenesis in human glioblastoma

**Figure 3:**
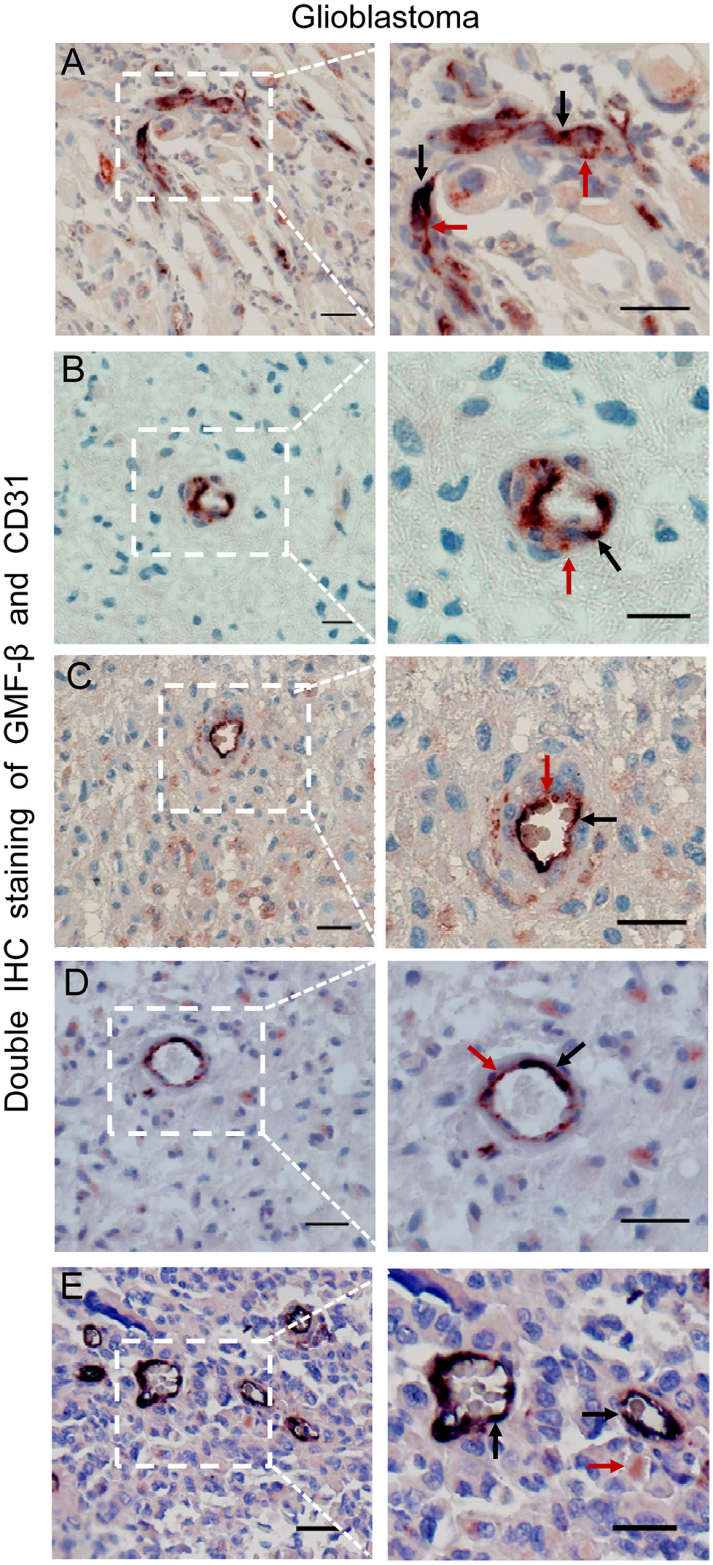
GMF-β is involved in neovasculogenesis in human glioblastoma **A**. Co-expression of GMF-β and CD31 in several tumor cells. **B**. The GMF-β^+^/CD31^+^ incomplete microvessel-like structure in hypovascular zones. **C**. GMF-β^+^/CD31^+^ immature microvessel in hypovascular zones. **D**. Single GMF-β^+^/CD31^+^ mature microvessel in vascularized areas. **E**. GMF-β^−^/CD31^+^ mature microvessels in vascularized areas. Red arrows denote GMF-β staining, black arrows indicate CD31 staining. Scale bar: 50 μm.

Immunohistochemical double staining for GMF-β and CD31 was applicated to further observe the relationship between GMF-β expression and neovascular pattern in all glioma specimens. Co-expression of GMF-β and CD31 was scarcely found in microvascular endothelia of low-grade glioma. Considerably massive dual-staining of GMF-β and CD31 in microvascular endothelia was found in high-grade glioma. Interestingly, in hypovascular zones of glioblastoma (GBM) tumor core, CD31 expression was detected in some GMF-β-positive tumor cells (Figure 3A), indicating an endothelial phenotype of these anaplastic cells. Moreover, dual-labeled GMF-β and CD31 were observed in some incomplete microvessel-like structures (Figure 3B) and immature microvessels (Figure 3C) in GBM tumor cores, inferring the endothelialization and vasculogenic activity of these GMF-β-positive GBM cells. The mature microvessels dually expressing GMF-β and CD31 were found in vascularized areas of high grade gliomas (Figure 3D), indicating a tumoral origin of these glioma vessels. We also noticed some completely matured microvessels that were stained by CD31 only. No expression of GMF-β was observed in this kind of microvessel (Figure 3E). Thus, a complete picture was illustrated, regarding the role of GMF-β in glioma neovascularzation. These phenomena implied that over-expression of GMF-β in GBM cells could trigger neovasculogenesis initiating from an endothelialization process of malignant glioma cells.

### GMF-β knockdown inhibits the proliferation and tubulogenesis of U87 cells *in vitro*

**Figure 4:**
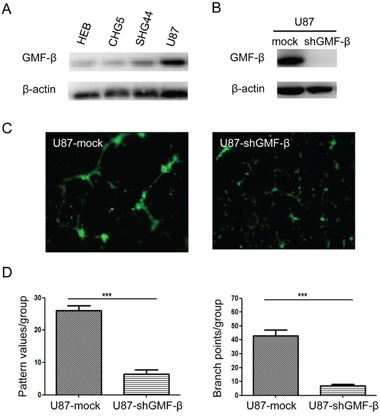
Tubulogenesis of human U87 glioblastoma cells is inhibited by GMF-β knockdown **A**. Protein levels of GMF-β in human glial cell line (HEB) and human glioma cell lines of the different grades (CHG5, SHG44, U87). **B**. Assessment of GMF-β knockdown in U87 cells by western blotting. **C**. Tube formation by U87 mock cells (left panel); impaired tube formation by U87-shGMF-β cells (right panel). Inspection under a phase contrast fluorescent microscope (× 100). **D**. Quantified tubulogenesis of U87 mock cells and U87-shGMF-β cells by pattern recognition system (left panel) and branch point counting system (right panel).*** indicates significant difference with *P* < 0.001.

Protein levels of GMF-β in human normal glial cell line, HEB, as well as different grades of human glioma cell lines (CHG5, SHG44, U87) were evaluated by western blots. Similar to the results in glioma tissue, GMF-β protein levels corresponded with malignancy of glioma cell lines. High-level of GMF-β expression was detected in highly malignant GBM cell line U87 (Figure 4A). Therefore, U87 cells were selected for further experiments. GMF-β was knocked down by shRNA and the knockdown efficiency in U87-shGMF-β cells was assessed by western blotting (Figure 4B).

Then we investigated the effect of GMF-β knockdown on the proliferation and tubulogenesis of U87 cells *in vitro*. We found that GMF-β knockdown inhibited U87 cell proliferation (*P* < 0.05; Supplementary Figure S1). This finding was consistent with the positive correlation between GMF-β expression and Ki67 index from the clinicopathological analysis mentioned above (Table 2). The tubulogenic capacity of U87-mock and U87-shGMF-β cells was evaluated. In our experimental condition, tubulogenesis was initiated in U87 cells, extremely relying on endothelial culture medium (Supplementary Figure S2A). No tubules were formed by U87 cells cultured in routine culture medium (Supplementary Figure S2B). GMF-β knockdown dramatically impaired tubulogenesis in U87 cells. Tube formation of U87 mock cells was fully developed by 18–20 hours (Figure 4C, left panel). In contrast, impaired tubulogenesis was presented in U87-shGMF-β cells that began to form rare tubules by 28 hours (Figure 4C, right panel). Tube formation in each group was quantified by pattern recognition (Figure 4D, left panel) and branch point counting system (Figure 4D, right panel), respectively. According to the two assessment system, tubulogenesis of U87-shGMF-β cells was shown to be significantly decreased when compared with that of U87 mock cells (*P* < 0.001).

### GMF-β knockdown represses the formation of human CD31-positive microvessels in murine orthotopic glioma model

**Figure 5:**
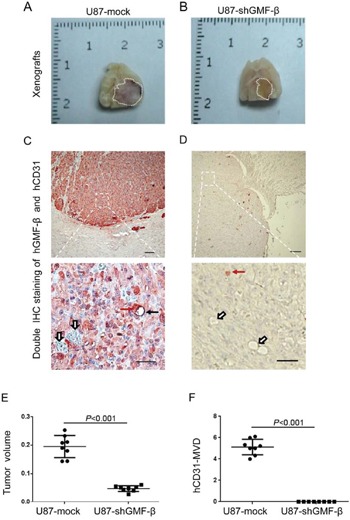
GMF-β knockdown suppresses tumor growth and the formation of human-CD31 positive microvessels (hCD31-MVs) in orthotopic U87 xenograft models **A**. The cross-sectional appearance of xenografted gliomas from U87-mock group **B**. The cross-sectional appearance of xenografted gliomas from U87-shGMF-β group. Gross tumor boundaries were delineated by white dotlines. **C**. A hCD31-microvessel and several hCD31-negative vessels in U87-mock tumor. **D**. No hCD31-MVs, only hCD31-negative vessels in U87-shGMF-β tumor. Red solid arrows indicate hGMF-β staining in tumor cells, black solid arrows show hCD31 staining on microvasular endothelia, and open arrows denote vessels negative for hCD31. Scale bar: 100 μm in upper pannels; 50 μm in lower pannels. **E**. Quantitative comparisons of gross tumor volume between U87-mock and U87-shGMF-β group. **F**. Quantitative comparisons of hCD31-microvessel densities (MVDs) between U87-mock and U87-shGMF-β group.

To further determine the effect of GMF-β on tumor neovasculogenesis *in vivo*, an orthotopic glioma model was used. The severe combined immunodeficient (SCID) mice were intracranially implanted with U87-shGMF-β cells, or U87-mock cells, respectively. No weight loss and neurological signs were observed in SCID mice of U87-shGMF-β group up to week 5. In the U87-mock group, all mice presented with neurological symptoms at week 4. The xenografted glioma specimen were obtained postmortem from U87-mock group and U87-shGMF-β group (Figure 5A and 5B).

All xenografted glioma specimens, as well as their mouse peritumoral brain tissue, were subjected to immunohistochemical double-staining for human-GMF-β and human-CD31. In xenografted glioma specimens of U87-shGMF-β group, there were only a few scattered distribution of GMF-β in tumor cells, showing the actual GMF-β knockdown effect (Figure 5D). In peritumoral mouse brain tissue, we did not find any blood vessel stained by the anti-human CD31 antibody. In contrast, murine vasculature could be stained by an anti-murine CD31 antibody (not shown), indicating the specificities of the human and murine CD31 antibodies. Human CD31-positive microvessels were detected in U87-mock xenograft tumors (Figure 5C), but not found in U87-shGMF-β xenograft tumors (Figure 5D). The xenograft tumor volumes in U87-shGMF-β group were significantly smaller than those in U87-mock group (*P* < 0.001; Figure 5E). Furthermore, human CD31-positive microvessel densities (hCD31-MVDs) in U87-shGMF-β xenograft tumors were significantly decreased in contrast to U87-mock xenograft tumors (*P* < 0.001; Figure 5F). These results indicated the indispensable role of GMF-β in inducing tumor endothelial cells (TECs) derived from malignant glioma cells.

## DISCUSSION

Tumor neovessels are characterized by striking abnormalities in structure and function, which contributes to tumor propagation and therapy resistance in glioma, especially in glioblastoma [3, 27]. A variety of classic pro-angiogenic factors, including VEGF, HIF-1, and CXCR4, have been reported to be regulators for the formation of glioma neovessels [10, 28]. Therapeutics based on blocking tumor angiogenesis have reached the clinic, but resulted in limited efficacy. Recently, growing evidences have shown vasculogenic potential in highly plastic tumor cells, such as glioblastoma cells, melanoma cells, hepatocellular carcinoma cells and breast cancer cells [29–31]. Therefore, targeting vasculogenic potential of tumor cells will open new avenues for developing anti-vasculogenic therapy in glioma [32, 33].

GMF-β was once regarded as a neural lineage-specific factor [19]. However, during gliomagenesis, the roles of GMF-β have not yet been clearly elucidated. In this study we report, for the first time, that GMF-β is expressed not only in glioma cells, but also in some microvascular endothelia. The clinicopathological analysis revealed the correlations of elevated GMF-β expression with high tumor grade, high MVD and poor prognosis in human glioma. These phenomena imply that GMF-β can promote glioma progression, probably due to its pro-vasculogenic potential.

To explore the essence of these phenomena, we reinvestigated glioblastoma specimens with dual-labeling of GMF-β and CD31. CD31 as a marker of endothelial lineage, is used primarily to demonstrate the presence of endothelial cells. Interestingly, we observed that, some glioblastoma cells co-expressed GMF-β and CD31. Physiologically, normal astrocytes do not express CD31. CD31 expression in glioblastoma cells that derived from malignantly transformed astrocytes, implied the endothelialization of such neoplastic cells [11]. Moreover, co-expression of GMF-β and CD31 was detected in some incomplete microvessel-like structures, immature and mature microvessels. Therefore, participation of GMF-β in glioma neovasculogenesis was illustrated.

We further verified that GMF-β is an inducer for vasculogenic activity of glioma cells, not merely a glial lineage marker. A matrigel-based tube formation assay has been widely applied to assess vasculogenic activity of given cell lines *in vitro* [34]. As expected, tubulogenic capacity of glioblastoma U87 cells was mitigated via GMF-β deprivation. In addition, our results from a pilot experiment showed that, tubulogenesis of U87 cells relied on the stimulation of some proangiogenic factors in the conditioned medium. More excitingly, human CD31-positive microvessels were detected in U87-derived xenograft tumors. It indicated that these microvessels were originated from human U87 glioblastoma cells. GMF-β expression in U87 cells was also shown to be indispensable for the formation of tumor cell-derived neovessels in murine models. These discoveries were strongly in favor of our histopathologic findings in human glioblastoma specimens. According to the *in vitro* and *in vivo* results, we conclude that vasculogenic potential of glioblastoma cells is activated in a GMF-β-induced and microenvironment-dependent pattern.

Interestingly, GMF-β expression in tumor endothelial cells (TECs) was proved to be an independent predictor of survival in patients with glioma. It means that glioma tumor cells can serve as an important complement to form neovessels, Tumor cell-derived neovessels may be closely associated with the aggressive behavior in glioma. In one word, the ratio of endothelial transdifferentiation in glioblastoma cells could be a crucial checkpoint for glioma progression. In contrast, GMF-β expression level in tumor cells was not an independent prognostic factor. Impact of tumoral GMF-β expression on prognosis might be affected by other clinicopathologic factors, which could infer the manifold functions of GMF-β in glioma cells.

Related studies mainly focused on GMF-β's roles in promoting glial differentiation of astrocytes and glioma cells [20]. However, the glial differentiation activity of GMF-β in astrocytoma was found to be much weaker than in normal brain [35]. Our study also suggested that, endogenous GMF-β should predominantly act to induce endothelialization of tumor cells in malignant glioma. Lim *et al* [21] found that GMF-β could promote the initial growth, but also restore contact inhibition of rat C6 glioma cells and human HG-1 glioma cells *in vitro*. They also demonstrated the growth-suppressive effect of GMF-β on C6 glioma cells subcutaneously implanted into athymic mice. In our study, GMF-β has shown to be indispensable to maintain *in vitro* proliferative activity and *in vivo* tumorigenicity of U87 glioblastoma cells. Our discoveries are partially contradicted with other related reports on GMF-β. The discrepancy between results of ours and others may be due to the difference in experimental methods, such as cell lines, culture conditions, especially animal models of tumorigenesis. The *in-situ* condition where glioma grows up can not be well reproduced in a subcutaneous xenograft model. The glioma microenvironment can be better simulated in the orthotopic xenograft model we adopted.

Recently, advances have been made in researches on GMF-β. It is reported to be an adaptive protein whose ultimate effect depends on the environmental context [20]. In context with all findings from other research groups and ours, we conclude that GMF-β might induce glioblastoma cells to differentiate into both glial lineage and endothelial lineage. Our conclusion is consistent with these findings which revealed the effect of a microenvironmental “niche” on multidirectional differentiation of plastic cells and multifunction of regulatory proteins *in vivo* [36, 37]. More attention should be paid to the cell type-specific modulation of tumor neovasculogenesis in tissue microenvironment.

Taken together, we innovatively found the specific expression pattern of GMF-β in glioma cells and TECs, and evaluated their correlation with tumor grade, MVD, and clinical prognosis. Furthermore, we demonstrate that GMF-β, an endogenous cytokine in neural lineage cells, could be an inherent provasculogenic factor in inducing glioma cell-derived neovessels. Our findings may offer a prognostic biomarker as well as a cell type-specific target for anti-vasculogenic therapy in glioma. In future study, we will further elucidate GMF-β-induced signaling pathways for glioma neovasculogenesis.

## MATERIALS AND METHODS

### Tissue specimens and patient characteristics

One hundred and forty-six cases of glioma specimens were surgically obtained from Southwest Hospital (affiliated to Third Military Medical University, Chongqing) from January 2006 to December 2009. Pathological diagnoses were confirmed by three neuropathologists independently according to the 2007 WHO classification of central nervous system tumors. Normal brain tissues of 10 patients dying from non-cerebrovasular diseases were obtained by autopsy and served as controls.

The clinical features of these cases have been summarized in Supplementary Table S2. All patients with complete information were followed up after operation until September 20th, 2012, with a median follow-up time of 16.8 months for progression-free-survival and 19.5 months for overall survival. Written informed consents for the biological studies were obtained from the patients or their guardians. All of the above processes were complying with the principles of Helsinki Declaration and approved by Ethics Committee of Third Military Medical University.

### Immunohistochemistry (IHC)

The formalin-fixed, paraffin-embedded (FFPE) samples were sectioned for IHC. The Envision Peroxidase/DAB Detection System kit (K5007; Dako, Glostrup, Denmark) was used for single immunostaining of GMF-β, developing brown color. The DouSP™ double-staining kit (KIT-9999; Maixin-Bio, Fuzhou, China) was applied to double-staining of GMF-β and CD31, developing red color with streptavidin-peroxidase-conjugated 3-Amino-9-ethylcarbazole (AEC), and black-purple color with streptavidin-alkaline-phosphatase-conjugated 5-bromo-4-chloro-3-indolyl phosphate (BCIP) / nitro blue tetrazolium (NBT). The primary antibodies were as follows: rabbit anti-human GMF-β polyclonal antibody (NBP1–89755, dilution1:200; Novus-bio USA) and mouse anti-human monoclonal CD31 antibody (NB600–562, dilution 1:10; Novusbio, USA). Isotype controls were used for the two primary antibodies, including rabbit immunoglobulin (IgG) for GMF-β and mouse IgG1κ for CD31.

IHC expression of GMF-β was quantified separately for glioma cells and microvessel endothelia [38]. The immunoreactive staining of GMF-β in tumor tissue was scored by applying a semi-quantitatively immunoreactive scoring (IRS) system. Category A documented the intensity of immunostaining as 0 (no immunostaining), 1 (weak immunostaining), 2 (moderate immunostaining), and 3 (strong immunostaining). Category B documented the percentage of immunoreactive tumor cells or microvessels as 0 (none), 1 (< 25%), 2 (26–50%), 3 (51–74%), and 4 (> 75%). Multiplication of category A and B resulted in an IRS ranging from 0 to 12 for each tumor. We then used several grouping algorithms (IHC scores) in the tumor cells and endothelia (negative [-, IRS 0], weak positive [+, IRS 1–4], moderate positive [++, IRS 5–8] and strong positive [+++, IRS 9–12]) for further experiments. Microvessel density (MVD) was assessed in accordance with the criteria of Weidner [39]. Both IHC score and MVD counting were performed by three pathologists in a double-blinded manner. Images were captured and analyzed using Olympus BX51 microscope (Olympus, Tokyo, Japan) fitted with the Image-Pro Plus 6.0 software (Media Cybernetics, SilverSpring, MD, USA).

### Cell line and cell culture

Human glioma cell lines U87 (WHO Grade IV) were obtained from the American Type Culture Collection (ATCC) (Manassas, VA). The other two human glioma cell lines included SHG44 (WHO Grade III) and CHG5 (WHO Grade II). SHG44 was obtained from the Laboratory of Brain Tumors, Suzhou University (Suzhou, China). CHG5 was established in Institute of Pathology and Southwest Cancer Center (Chongqing, China). Human normal glial cell line, HEB, was generously provided by Professor Guang-mei Yan (Department of Pharmacology, Sun Yat-sen University, Guangzhou, China). All the cells were maintained in Dulbecco's Modified Eagle Medium (DMEM) containing 10% fetal bovine serum (FBS) (Gibco, Grand Island, NY, USA) and incubated at 37°C with 5% CO_2_ / 95% air.

### Western blotting

Western blotting was carried out routinely. The primary antibodies included rabbit anti-human GMF-β (NBP1–89755, dilution 1:200; Novus Biologicals, Littleton, CO, USA) and rabbit anti-human β-actin (#5125, dilution 1:1000; Cell Signaling, Boston, MA, USA). Protein bands were visualized with enhanced chemiluminescence (Amersham, Piscataway, MA, USA) following manufacture's instruction. GMF-β expression level was normalized to β-actin expression for each sample.

### GMF-β knockdown by shRNA

GMF-β-targeting short hairpin RNA (shRNA) sequences were designed using Invitrogen online BLOCK-iT RNAi Designer (http://www.invitrogen.com/RNAi). The sequence of shRNA targeting GMF-β was 5′-CCGGGAAGAATGGTTACGTGAGA AACTCGAGTTTCTCACGTAACCATTCTTCTTTTT G-3′. A nonsilencing sequence (5′-CCGGCGCTTCATTGT GTATAGTTATCTCGAGATAACTATACACAATGAAG CGTTTTTG-3′) was used for control. The GMF-β-targeting shRNA or mock fragment was inserted into the pMagic4.11eGFP-expressing vector (Sunbio, Co., Ltd., Shanghai, China). GMF-β-shRNA-expressing cells were selected and enriched by flow cytometry (BD FACSAria II).

### Tumor cell proliferation assay

U87-shGMF-β or U87-mock cells (1000 cells/100 μl culture medium) were seeded into 96-well plates. Cell numbers were counted every day for 6 days. At each interval, 10 μl cell counting Kit-8 solution (C0038; Beyotime, Shanghai, China) was added to each well and incubated for 2 hours at 37°C. The absorbance value at 450 nm was measured by Thermo Multiskan Spectrum Reader (Thermo Scientific, MA, USA). All treatments were performed in triplicate.

### *In vitro* angiogenesis assay

*In vitro* angiogenesis assay was performed and evaluated according to the instructions of *in vitro* Angiogenesis Assay Kit (ECM625; Millipore, Billerica, CA, USA). U87-shGMF-β or U87-mock cells (5 × 10^3^ per well, 96 well size) were seeded on the polymerized Matrigel, and incubated in Endothelial Cell Basal Media (EBM-2) supplemented with Endothelial Cell Growth Supplements (EGMTM-2; LONZA, Walkersville, MD, USA). Then, tube formation in each group was inspected under a phase contrast fluorescent microscope (BX51 CKX41-F32FL, Olympus, Tokyo, Japan).

The formation of tubules is a dynamic process, starting with cell migration and alignment, followed by the development of capillary tubes. The quantification of tubulogenesis followed two evaluation criterions, “pattern recognition” and “branch point counting”. According to “pattern recognition” evaluation, a numerical value was assigned to each pattern: 0, individual cells; 1, a queue of cells; 2, capillary tubes; 3, sprouting capillaries; 4, closed polygons; 5, complex mesh like structures. Thus, a numerical summation was scored in each view-field. By the criterion of “branch point counting”, the newly formed branch was defined as an isolated cellular line sprouting from existing capillary tubes. Branch points were counted and totaled in each view-field. Five random view-fields per well were observed at 100 × magnification, then the quantification values were averaged in each group.

### Murine orthotopic glioma model

The animal experiment was approved by Animal Ethics Committee of Third Military Medical University. The 6-week-old male severe combined immunodeficient (SCID) mice were provided by Experimental Animal Center of Third Military Medical University. These mice were evenly apportioned into two groups (*n* = 8). Each mouse was anesthetized and intracranially injected with 1 × 10^5^ U87-shGMF-β or U87-mock cells in 5 μl PBS. They were monitored daily for weight change, neurological signs and survival. All SCID mouse brains were collected postmortem and then sampled for immunohistochemistry. Gross tumor volume (Tv) was calculated according to the formula: “*Tv* = L (length) × W^2^ (width) / 2” [40].

### Statistical analysis

The linear regression model was adopted to measure the relationship between GMF-β expression level and MVD. Kaplan-Meier survival plots and log-rank statistics were used for comparison of survival rate. The Cox's proportional hazard model was applied for univariate and multivariate survival analysis. Pearson χ^2^ test was applicated to analyze the relationship between GMF-β expression and clinicopathological parameters. The optimal cut-point of GMF-β expression was determined by X-tile software (Version 3.6.1, Yale University, New Haven, CT) [41]. When two groups were compared, the Mann-Whitney test, unpaired or paired Student's t test were used. Data were expressed as the mean ± standard deviations (SD). All statistics were analyzed by GraphPad Prism 5.0 software. Statistic significance was assigned at *P* < 0.05.

## SUPPLEMENTARY TABLES AND FIGURES


