# Supplementary material for: Expressions of glia maturation factor-β by tumor cells and endothelia correlate with neovascularization and poor prognosis in human glioma
Source: Oncotarget. 2015 Oct 26;7(52):85750–63. doi: 10.18632/oncotarget.5509 (PMC5349871; doi:10.18632/oncotarget.5509)
Supplement: Supplementary file 1 [file oncotarget-07-85750-s001.pdf]

Expressions of glia maturation factor-β by tumor cells and endothelia correlate with neovascularization and poor prognosis in human glioma

SUPPLEMENTARY TABLES AND FIGURES

Supplementary Table S1: Univariate analyses of progression-free survival and overall survival in glioma patients

Factors	*Progression-free survival*		*Overall survival*	
	HR (95% CI)	*P*-*value*	*HR (95% CI)*	*P*-value
WHO grade	4.680 (3.252–6.735)	0.000	4.967 (3.340–7.385)	0.000
Gender	0.924 (0.631–1.352)	0.683	0.893 (0.606–1.316)	0.567
Age	1.056 (1.038–1.074)	0.000	1.058 (1.040–1.077)	0.000
KPS	0.981 (0.969–0.993)	0.002	0.984 (0.971–0.996)	0.008
Ki-67	2.876 (2.293–3.608)	0.000	2.786 (2.244–3.458)	0.000
Resection extent	0.544 (0.369–0.802)	0.002	0.518 (0.348–0.771)	0.001
Chemotherapy	0.529 (0.350–0.800)	0.003	0.501 (0.327–0.769)	0.002
Radiotherapy	0.341 (0.211–0.549)	0.000	0.364 (0.225–0.588)	0.000
Predominant side	1.194 (0.854–1.670)	0.299	1.224 (0.868–1.725)	0.249
Predominant lobe	0.965 (0.848–1.099)	0.593	0.982 (0.860–1.122)	0.792
MVD	1.064 (1.046–1.082)	0.000	1.068 (1.049–1.087)	0.000
GMF-β (in endothelia)	1.403 (1.315–1.497)	0.000	1.423 (1.331–1.521)	0.000
GMF-β (in tumor cells)	1.154 (1.088–1.224)	0.000	1.170 (1.102–1.241)	0.000

Abbreviations: HR, Hazard ratio; CI, Confidence interval; KPS, Karnofsky performance status; MVD, Microvessel density.

Supplementary Table S2: The clinical features of 146 patients with glioma

Feature	*WHO Grade*	
	Low grade (n = 46)	*High grade (n = 100)*
Age (Years)		
≥45	4	62
<45	42	38
Gender		
Male	29	60
Female	17	40
Predominant side		
Left	19	47
Right	26	49
Middle	1	0
Doube	0	4
Predominant lobe		
Frontal	26	43
Temporal	7	39
Parietal	3	9
Occipital	0	4
Other	10	5
KPS		
≥80	33	52
<80	13	48
Resection extent		
GTR	28	36
PR	18	64

Abbreviations: KPS, Karnofsky performance status; GTR,Gross total resection; PR, Partial resection.

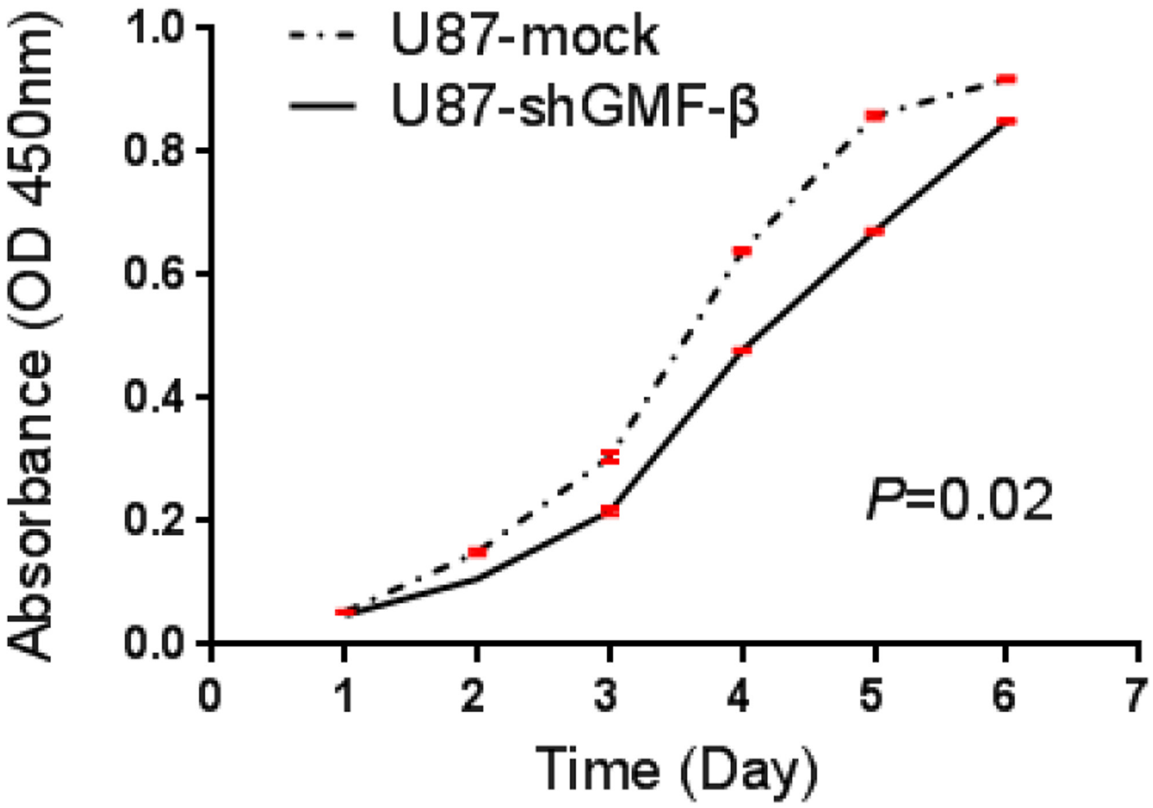

Supplementary Figure S1: Inhibition effect of GMF-β knockdown on U87 glioma cell proliferation. Growth curves of U87-shGMF-β cells and U87-mock cells obtained by cell proliferation assay.

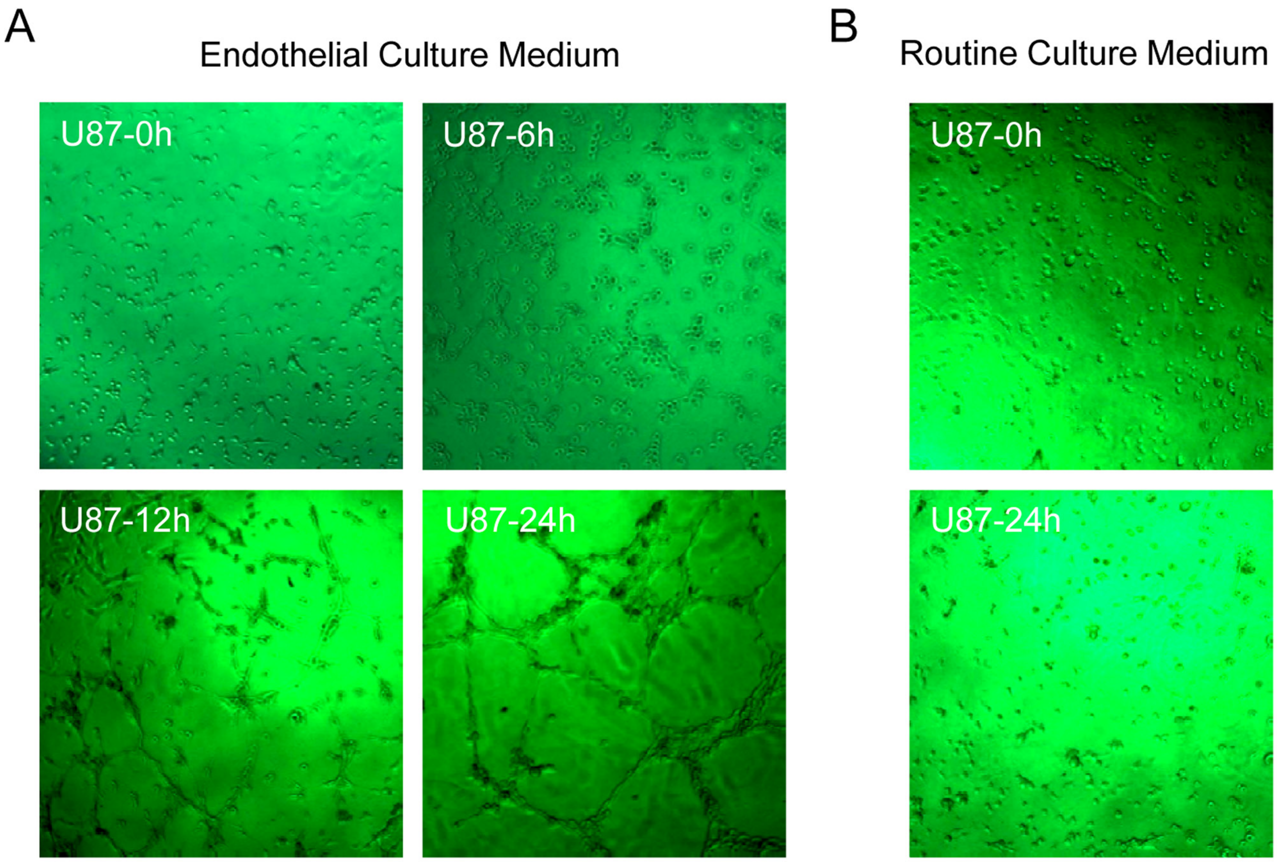

Supplementary Figure S2: U87 cell-induced tubules in a culture medium-dependent manner. A. U87 cell-induced tubules were observed in endothelial culture medium. **B.** No tubules were formed by U87 cells cultured in routine culture medium.
